# Donating a Kidney to a Stranger: Are Healthcare Professionals Facilitating the Journey? Results From the BOUnD Study

**DOI:** 10.3389/ti.2023.11257

**Published:** 2023-05-30

**Authors:** Hannah Maple, Petrut Gogalniceanu, Rebecca Gare, Lisa Burnapp, Heather Draper, Joseph Chilcot, Sam Norton, Nizam Mamode

**Affiliations:** ^1^ Department of Transplantation, Guy’s and St Thomas’ NHS Foundation Trust, London, United Kingdom; ^2^ UK and NHS Blood and Transplant, Bristol, United Kingdom; ^3^ Health Sciences, Warwick Medical School, University of Warwick, Coventry, United Kingdom; ^4^ Department of Psychology, Institute of Psychiatry, Psychology and Neuroscience, King’s College London, London, United Kingdom

**Keywords:** transplantation, kidney donation, living, unspecified, altruistic

## Abstract

Unspecified kidney donors (UKDs) are approached cautiously by some transplant professionals. The aim of this study was to interrogate the views of UK transplant professionals towards UKDs and identify potential barriers. A purposely designed questionnaire was validated, piloted and distributed amongst transplant professionals at each of the 23 UK transplant centres. Data captured included personal experiences, attitudes towards organ donation, and specific concerns about UKD. 153 responses were obtained, with representation from all UK centres and professional groups. The majority reported a positive experience with UKDs (81.7%; *p* < 0.001) and were comfortable with UKDs undergoing major surgery (85.7%; *p* < 0.001). 43.8% reported UKDs to be more time consuming and 52% felt that a mental health assessment should take place before any medical tests. 77% indicated the need for a lower age limit. The suggested age range was broad (16–50 years). Adjusted mean acceptance scores did not differ by profession (*p* = 0.68) but higher volume centres were more accepting (46.2 vs. 52.9; *p* < 0.001). This is the first quantitative study of acceptance by transplant professionals to a large national UKD programme. Support is broad, however potential barriers to donation have been identified, including lack of training. Unified national guidance is needed to address these.

## Graphical Abstract

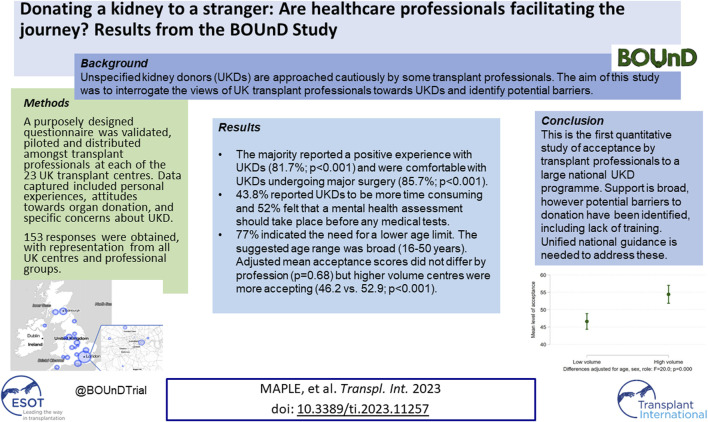

## Introduction

Unspecified kidney donation (UKD), also known as altruistic or non-directed altruistic donation, describes the intention of an individual to donate a kidney to a stranger during their lifetime [1]. UKD has significant potential for patient benefit by contributing to organ sharing schemes which facilitate transplants between blood group and human leucocyte antigen (HLA) incompatible pairs. In the 2019/20 financial year, 95 unspecified kidney donors (UKDs) in the United Kingdom (UK) (10% of living donors) facilitated 146 living donor kidney transplants. Critically, 48 UKDs donated directly to high-priority candidates on the waiting list and 47 initiated Altruistic Donor Chains (ADCs) as part of the UK Living Kidney Sharing Scheme (UKLKSS) [2, 3].

Despite a measurable benefit to the UK kidney transplant programme, public endorsement [4, 5], and comparable clinical outcomes to specified kidney donors (SKDs) [6, 7], there is historic evidence of UKDs being approached with a degree of caution and suspicion by some transplant professionals [8]. Qualitative studies have shown that UKDs detect these negative attitudes during clinical encounters, and these manifest as attempts to discourage donation and the presentation of inconsistent information. Donors have also reported feeling distressed at the mandatory requirement for a mental health assessment [9, 10], which is partly based upon the desire to exclude underlying psychopathological motives. This makes donors feel overly scrutinised and as though they must prove their sanity [11].

Despite the issues mentioned above, there is no existing evidence that quantifies the attitudes of UK transplant professionals towards UKD and whether these are consistent with what has been reported by UKDs. Additionally, since the beginning of the UKD programme there have been large centre variations across the UK. Currently 45% of UKD donations take place in just six out of 23 transplant centres [6]. Of the six, three are in the south of England. There is no robust explanation for these variations, nor whether this is a manifestation of the professional attitudes and values of the transplant professionals working within these centres.

The Barriers and Outcomes in Unspecified Donation (BOUnD) study was devised to conduct a comprehensive assessment of the UK UKD programme. BOUnD is a mixed methods study aiming to capture clinical and psychosocial data on outcomes following UKD (and how these compare with Specified Kidney Donation (SKD)), as well as data on the attitudes of transplant professionals towards UKD [12]. The study arm investigating transplant professionals’ attitudes consists of two components. The qualitative arm involved interviews with transplant professionals from high and low volume UKD centres. The quantitative arm captured data from professionals across the country using a validated questionnaire. These were both informed by focus groups held with both service users and transplant professionals. This paper presents the results from the quantitative study, the aim of which was to elicit the views of UK transplant professionals towards different aspects of UKD, and whether any of these could be identified as potential barriers to donation. We hypothesised that:

1. A minority of transplant professionals would express negative views toward unspecified kidney donation and unspecified kidney donors
2. Surgeons and nephrologists working with unspecified kidney donors would hold more negative views than nursing and other clinical staff
3. Individuals working in low volume centres would hold more negative views than those working in high volume centres; potentially providing a contributory reason for why donation rates are lower in these centres

## Materials and Methods

### Study Design

We undertook a cross-sectional survey of transplant professionals from across the UK (Supplementary Digital Content File (SDC) S1). A focus group was held with former service users and transplant professionals to inform the themes to be captured. The questionnaire was subsequently written, undergoing multiple iterations which were circulated amongst the research team. Once this was finalised the questionnaire underwent further refinement and reliability testing, as well as robust validation and piloting. The details of this are documented in Supplementary Digital Content File S2.

Transplant professionals were defined as any patient-facing healthcare worker involved in the care of a potential unspecified kidney donor. This included surgeons and nephrologists, ward and outpatient nurses, donor co-ordinators, independent assessors, psychiatrists, and clinical administrative staff. Physicians and surgeons were only recruited if they were at consultant or senior trainee level, having declared transplantation as a specialist interest. The rationale for this was to reduce the potential for bias within the sample by only including those with sufficient clinical experience in living donation.

The principal investigator and nominated transplant co-ordinator at all of the 23 UK transplant centres were charged with distributing electronic or paper-based questionnaires. A subsequent snowball sampling approach was encouraged to optimise recruitment of relevant individuals both within and outside their organisation. Relevant professionals outside the transplant centre include those working within non-transplant renal centres who undertake their own UKD workup locally before referring them to the transplant centre for surgery. Due to the large variation in transplant centre size we aimed to have at least one clinician from each professional group from each centre. Interim analysis of the results at 6 months identified low-responding centres and professional subgroups, leading to recruitment drives targeting these groups. Adequate representation was agreed to have been achieved once one clinician from each professional group in each transplant centre had completed the questionnaire.

### Statistical Analysis

Categorical variables were described as the number of non-missing values and percent. Continuous variables were described using means and standard deviations, or medians and quartiles where high levels of skew were observed. Differences between variables across groups for continuous variables were assessed using mixed effects models, including a random intercept to account for the nesting of individuals within centres. Robust standard errors were estimated, and the 5% alpha level used for interpreting *p*-values.

Responses to some items were combined to form scales indicating the level of acceptance of UKD. A pool of 13 items potentially indicating acceptance of UKD were selected by the research team and the suitability for generating an acceptance score was confirmed by exploratory factor analysis. Specifically, 7 items loaded onto an acceptance factor were retained as an acceptance score (Cronbach’s alpha of 0.77). To account for differing response categories across items the scale of the score was standardised with the mean for the sample set at 50 and the standard deviation of 10. A higher score indicated greater acceptance of UKD. Whilst arbitrary, it allowed for comparisons across groups within the sample. Analyses were conducted in Stata 15.1 and IBM SPSS version 24. Full details, along with the psychometrics for the score, is provided in Supplementary Digital Content File S3.

## Results

Demographics and involvement with UKDs (Table 1).

**TABLE 1:** Sample demographics and involvement in Unspecified Kidney Donation.

	n (%)
Gender	
Male	57 (37.2)
Female	96 (62.7)
Age	
25–34	12 (7.8)
35–44	48 (31.4)
45–54	64 (41.8)
>55	29 (19)
Role	
Administrative staff	3 (2.0)
Inpatient nurse	11 (7.2)
Outpatient nurse	3 (2.0)
Co-ordinator	42(27.5)
Consultant Physician	28 (18.3)
Trainee Physician	5 (3.3)
Consultant Surgeon	28 (18.3)
Trainee Surgeon	8 (5.2)
Other Healthcare Professional	25 (16.2)
Member of minority ethnic group	
Yes	21 (13.7)
No	136 (82.4)
Prefer not to answer	6 (3.9)
Consider themselves religious	
Yes	39 (25.5)
No	109 (71.2)
Prefer not to answer	5 (3.3)
Consider themselves spiritual	
Yes	62 (40.5)
No	82 (53.6)
Prefer not to answer	9 (5.9)

**FIGURE 1:**
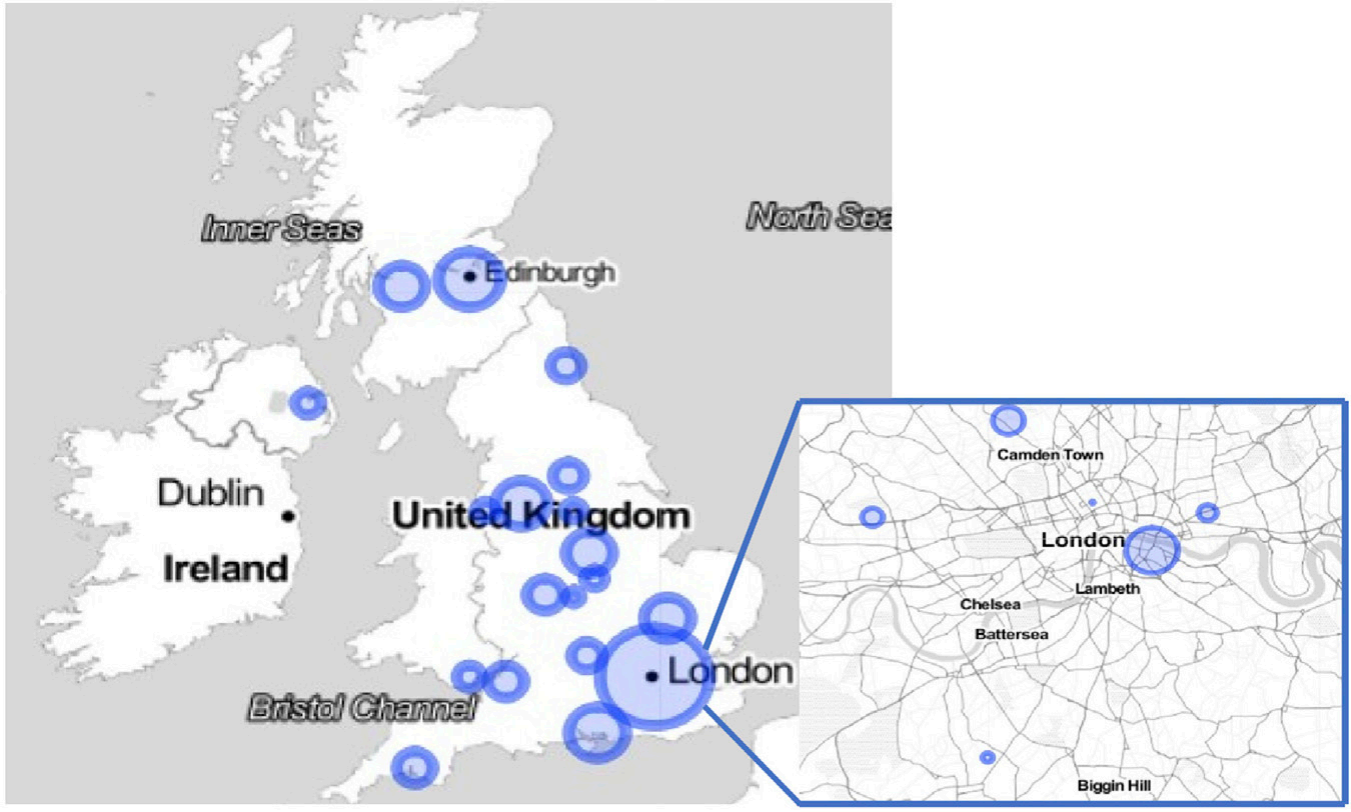
Map demonstrating distribution of participation across the UK.

The study provided a comprehensive coverage of the UK transplant community, covering 153 individuals from all 23 UK centres (Figure 1). The majority of participants were women (63%), and the most represented professional role was transplant coordinator (28%). Most participants were aged between 45 and 54 years and did not consider themselves to be from a minority ethnic group. A quarter considered themselves to be religious, with a slightly higher proportion identified as being spiritual (41%). Most respondents (77%) had between 2 and 10 years of experience in the field (77%) and 96 (64%) stated they have been involved with five or more UKDs.

Due to the snowball recruitment strategy, it was not possible to calculate a denominator of our sample size, as it is impossible to account for how many individuals received the questionnaire, nor how many individuals worked within the transplant programme within each centre. A surrogate marker was calculated based on the number of nephrologists, surgeons and co-ordinators responding to the questionnaire per centre; data obtained from the principal investigators at each site. This demonstrated a 73% response rate to the questionnaire amongst clinicians and a 68% response rate amongst transplant co-ordinators.

The questionnaire covered a range of topics pertinent to UKDs, the full range of which cannot be discussed at length as part of this manuscript. Those questions directly relevant to the hypotheses are provided below.

Hypothesis 1 A minority of transplant professionals would express negative views toward UKD / UKDs.

The majority of participants (81.7%) stated that their experience with UKDs had been generally positive, where a significance test against a null hypothesis of 50% was *p* < 0.001; CI 75.4%–87.7%. Similarly, the majority of participants (85.7%) said they were comfortable with UKDs undergoing major surgery, where a significance test against a null hypothesis of 50% was *p* < 0.001; CI 80.2%–91.2%. A considerable proportion of individuals did hold some negative views, including UKDs being more time consuming (43.8%; CI 35.9%–51.7%) and having a higher dropout rate (45.1%; CI 37.2%–53.0%) (Table 2).

Participants were specifically asked about their concerns about outcomes and motivations in UKDs (Table 3). High numbers of professionals felt that UKDs were more likely to have a history of mental health problems and expressed concerns for donors’ long-term mental and physical health, regret, and the potential for burden upon the donor’s family. This view was supported by the large numbers (83%; CI 77.0%–89.0%) stating that a formal mental health assessment should remain mandatory as part of the workup process. Of these, a small majority (52%; CI 43.3%–60.7%) felt this should take place before any medical tests.

Whilst UKD was broadly viewed positively, one area of significant contention was donor age. There was little consensus between participants about official upper and lower age limits for donation within their centre, with significant numbers unsure. Participants were asked separately whether they felt there ought to be age limits for UKD. Only 15% (CI 9.3%–20.7%) thought that an upper age limit should apply; and where this was indicated, this should be 70 years (range 50–80 years). More participants (77%; CI 70.3%–83.7%) thought a lower age limit should apply; and where this was indicated, should be 25 years (range 16–50 years).

A separate indication of negative feelings towards UKD was demonstrated in the responses to questions relating to whether the individual would consider being a living kidney donor themselves. A significantly higher proportion were comfortable with the idea of being a specified donor (86.9%), compared with only 21.6% comfortable with the idea of being an unspecified donor (*p* = 0.006).

**TABLE 2:** Professionals and UKDs.

	n (%)					
	Strongly agree	Agree	Neither agree nor disagree	Disagree	Strongly Disagree	
“I am confident dealing with people wishing to become UKDs”	65 (42.5)	69 (45.1)	15 (9.8)	3 (2.0)	1 (0.7)	
“My experience with people wishing to become unspecified (non-directed altruistic) donors has been generally positive”	48 (31.4)	77 (50.3)	25 (16.3)	3 (2.0)	0 (0)	
“I am comfortable with UKDs undergoing major surgery”	42 (27.5)	89 (58.2)	16 (10.5)	5 (3.3)	1 (0.7)	
Compared to SKDs BEFORE donation, potential UKDs:						
Have a higher dropout rate	7 (4.6)	62 (40.5)	57 (37.3)	27 (17.6)	0 (0.0)	
Are more time consuming for transplant professionals	14 (9.2)	53 (34.6)	42 (27.5)	40 (26.1)	4 (2.6)	
Need a greater number of assessments or investigations compared with specified living donors	2 (1.3)	44 (28.8)	45 (29.4)	59 (38.6)	3 (2.0)	
Compared to SKDs AFTER donation, potential UKDs:						
“UKDs receive less support after donation”	2 (1.3)	6 (3.9)	37 (24.2)	73 (47.7)	35 (22.9)	
More likely to seek medical help from transplant units regarding donation related issues	6 (3.9)	22 (14.4)	62 (40.5)	61 (39.9)	2 (1.3)	
More likely to seek mental health help regarding donation related issues compared to SKDs	2 (1.3)	15 (9.8)	72 (47.1)	62 (40.5)	2 (1.3)	
More likely to seek medical help from transplant units regarding non-donation related issues compared to specified donors	5 (3.3)	18 (11.8)	69 (45.1)	59 (38.6)	2 (1.3)	
	Much better	Better	Same	Worse	A lot worse	Unsure
“How are UKDs treated during the donation process compared with SKDs”	1 (0.7)	9 (5.9)	133 (86.9)	2 (1.3)	0 (0.0)	8 (5.2)

**TABLE 3:** Concerns about donation and donor motivations.

	n (%)				
	Strongly agree	Agree	Neither agree nor disagree	Disagree	Strongly disagree
“I am worried about the potential long-term effects of UKD on the donor’s…”					
Physical health	10 (6.5)	55 (35.9)	29 (19.0)	49 (32.0)	10 (6.5)
Psychological health	7 (4.6)	51 (33.3)	40 (26.1)	48 (31.4)	7 (4.6)
“I am worried UKDs may regret their decision to donate in the future”	3 (2.0)	47 (30.7)	46 (30.1)	51 (33.3)	6 (3.9)
“I am worried that UKD is potentially a burden for the donor’s family”	10 (6.5)	55 (35.9)	29 (19.0)	49 (32.0)	10 (6.5)
“I believe unspecified (non-directed altruistic) living kidney donors make balanced decisions when choosing/deciding whether to donate or not”	28 (18.3)	76 (49.7)	43 (28.1)	5 (3.3)	1 (0.7)
“I think many people wishing to be UKDs are more likely to be risk-takers who do not fully consider the consequences of their actions”	1 (0.7)	16 (10.5)	48 (31.4)	74 (48.4)	14 (9.2)
“I think many people wishing to be unspecified (non-directed altruistic) kidney donors are likely to have a history of mental health problems”	3 (2.0)	25 (16.3)	40 (26.1)	66 (43.1)	19 (12.4)
“I believe it is possible for unspecified (non-directed altruistic) donors to be motivated purely by the desire to help others”	61 (39.9)	80 (52.3)	9 (5.9)	3 (2.0)	0 (0)
How often do you think that altruistic donors are motivated by…					
“Personal psychological benefit”	32 (20.9)	101 (66.0)	14 (9.2)	5 (3.3)	1 (0.7)
“Desire to improve social status”	3 (2.0)	30 (19.6)	55 (35.9)	59 (38.6)	6 (3.9)
“Religious or spiritual beliefs”	18 (11.8)	82 (53.6)	41 (26.8)	11 (7.2)	1 (0.7)
“Civic duty and social responsibility”	15 (9.8)	91 (59.5)	31 (20.3)	16 (10.5)	0 (0)
“Personal psychological ill-health”	6 (3.9)	24 (15.7)	60 (39.2)	57 (37.3)	6 (3.9)

Hypothesis 2 Surgeons and nephrologists working with UKDs would hold more negative views than nursing and other clinical staff.

**FIGURE 2:**
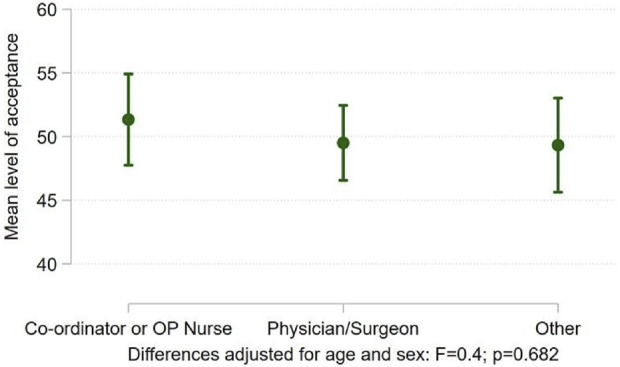
Differences in acceptance of UKD score between transplant professionals.

As described in the methods section, responses to some items were combined to form a scale indicating the level of acceptance of UKD. Figure 2 displays the mean acceptance scores for different categories of transplant professional. Adjusted means across groups were not statistically significantly different (*p* = 0.682), suggesting that professional background did not impact on UKD support or opposition. Levels of acceptance around UKD was unrelated to demographic variables. There were negative correlations between the score and more negative attitudes towards UKDs, including perceived resource use and decision making.

Hypothesis 3 Individuals working in low volume centres held more negative views than those working in high volume centres.

**FIGURE 3:**
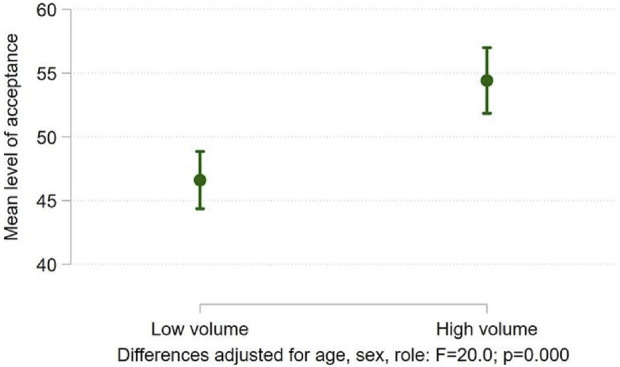
Differences in acceptance of UKD score between high and low volume centres.

The sample were divided into high and low volume centres. Six out of 17 centres were found to contribute to 50% of the total number of UKDs and these units were classed as high volume. Across the majority of questions there was no significant difference between the two groups in the way the questions were answered. Negative correlations were found with level of direct experience with UKDs, with those with less experience being less comfortable with UKD as a practice (*p* < 0.003) (Table 4). Conversely, fewer professionals in high volume centres felt that those making enquiries about UKD received a positive response (*p* < 0.001). They did not feel that staff at their centre had been adequately trained (*p* = 0.025), and nor did they feel that the facilities available were sufficient to support the UKD programme (*p* = 0.012). Fewer professionals at high volume centres reported positive experiences with UKD candidates (*p* < 0.001). Despite this, acceptance of UKD was significantly higher in high volume centres (46.2 vs. 52.9; *p* < 0.001) (Figure 3).

**TABLE 4:** Acceptance of UKD. Correlations between the acceptance score were calculated against a selection of variables from the questionnaire. Where items were both continuous, the correlation coefficient was estimated by the Pearson method. For ordinal and binary items the correlation coefficient was estimated by the polyserial method. Note that the Bonferroni adjusted critical value for *p* is reduced from *p* < 0.05 to *p* < 0.003. Acceptance scores were not related to demographic variables. They were, however, related to some variables relating to perceived resource use and more negative views regarding psychological motivations for wanting to donate.

	Acceptance score	
	Correlation	*p*-value
Age	−0.07	0.425
Female	0.03	0.749
Ethnic minority	0.07	0.551
Spiritual	−0.03	0.746
Religious	0.03	0.746
Altruism score	0.14	0.086
Direct experience with UKD	−0.51	0.000*
Years experience UKD	−0.41	0.000*
UKDs are likely to have mental health problems	−0.25	0.003
UKDs are more likely to be risk-takers	−0.26	0.002*
UKDs have a higher dropout rate	−0.07	0.434
UKDS are more time consuming	−0.14	0.118
UKDS need a greater number of assessments or investigations	−0.2	0.024*
UKDs more likely to seek medical help regarding donation issues	−0.19	0.029*
UKDs more likely to seek mental health help	−0.31	0.000*
UKDs more likely to seek medical help regarding non-donation issues	−0.26	0.003*
UKDs make balanced decisions when choosing to donate	0.53	0.000*
Personal psychological benefit	−0.03	0.744
Medical fitness	0.11	0.217

*p < 0.003.

## Discussion

This is the first quantitative study to report systematically on acceptance by transplant professionals to a large national UKD programme. We received responses from a broad range of different professionals involved in transplantation, with representation from each UK transplant centre. We hypothesised that negative views towards UKD would be held by a minority of transplant professionals, that surgeons and nephrologists would hold more negative views than nursing and other clinical staff, and that those working in low volume centres would have more negative views than those in high volume centres.

The study has shown that a large majority of transplant professionals are in support of UKD and that whilst levels of acceptance did not differ between professional groups, those from higher volume centres were more accepting. Whilst the majority of transplant professionals had positive experiences with UKDs, a considerable proportion perceived UKDs to be more time consuming with higher dropout rates. One of the aims of the prospective donor study being conducted as part of BOUnD [12] is to either confirm or deny these claims by providing prospective data on donor workup and donation times. Whilst formal analyses are ongoing, preliminary data has indicated that UKDs take longer to donate, but this is primarily due to their inclusion in the UKLKSS, which is conducted quarterly [13]. On occasions where UKDs donate to a high priority recipient on the waiting list, there is no significant difference in workup and donation times.

This study has confirmed long-held anecdotal views within the transplant community regarding donor motivations and concerns regarding mental health, both before and after donation. It is therefore understandable that the majority of participants wanted a formal, mandatory mental health assessment of UKDs to remain part of the workup process. This is in keeping with guidance from the British Transplantation Society, which considers it prudent for mental health assessments, conducted by a trained mental health professional, to remain best practice until more specific and sensitive evidence about the impact on mental health is available [9]. These guidelines are heavily influenced by a consensus statement written by transplant psychologists and psychiatrists on behalf of the European Association of Psychosomatic Medicine (EAPM) [14]. Data from this study has shown that a small majority of professionals felt that this assessment should take place prior to any medical tests being conducted. We believe this links two of the findings outlined above: the assumption of a higher incidence of mental health history within potential UKDs and the feeling that they are more likely to withdraw from the process. Should a transplant professional hold either or both of these views, it follows that by conducting mental health assessments early in the process, fewer individuals are subjected to further medical assessment, which is both costly and time consuming [15]. The EAPM recommend mental health screening “after initial medical screening, clinical assessment, and provision of information by the transplant team, but before any invasive investigations which carry physical risks,” in order to avoid subjecting potential donors to a risk of harm.

Transplant professionals with specific concerns related to potential UKDs withdrawing for mental health reasons may be reassured by findings from a qualitative study conducted as part of the BOUnD, which specifically investigated the experiences of UKD candidates who both completed and withdrew from the process [16]. In this study only very few participants not proceeding with UKD did so as a direct consequence of a mental health issues. Given that so many UKDs report difficulties with the experience of a mental health assessment [11] and that supply of adequately trained mental health professionals often leads to delays in the workup, concerns amongst about donors undertaking this assessment when they are towards the end of their work-up, may be misplaced.

A broader understanding of the attitudes of transplant professionals towards UKD can be gleaned from their own preferences regarding organ donation, with significantly more being comfortable with SKD compared to UKD. We postulate that this is due to an awareness and negative experiences of the risks of surgery which may only be deemed acceptable for a specified recipient. This is supported by previous research demonstrating that living donors are willing to accept significantly higher risks than transplant surgeons [17]. A qualitative interview study conducted in addition to this survey further probed some of these attitudes and the manuscript is currently under peer review.

An area of longstanding interest and controversy within UKD, and one which anecdotally has generated a huge amount of discussion amongst transplant professionals, is that of donor age. This study is the first to provide a quantitative assessment of transplant professionals’ views on this topic. As evident from the findings of this study, transplant professionals feel more strongly about a lower age limit than an upper age limit. Whilst there is no official lower limit for living donation in the UK, the BTS living donor guidelines [9] state that most programmes do not consider SKDs or UKDs aged under 18 years and view an age of 18–21 to be a relative contraindication to donation. The range of responses to what the lower age limit for UKD should be demonstrates the breadth of feeling within the transplant community. Proponents of donation later in life rationalise this viewpoint on the basis that time allows UKDs to live the majority of their lives with two kidneys (thereby reducing the long-term medical complications associated with donation) and to achieve an undefined degree of psychosocial maturity, which should in turn lead to lower levels of regret. Counterarguments against lower age limits are mainly focused on paternalism and whether this ought to override the autonomy of young people with capacity. There is no current evidence to prove that young people are more or less likely to regret their decision to donate, however there is evidence to show that younger donors (aged between 18 and 34) are more likely to develop end-stage renal disease and themselves require a transplant [9].

This study has highlighted a large gap in the literature which potentially fuels negative views and creates barriers towards UKD; a practice which has been proven to be of huge benefit patients with end-stage renal disease in the UK. In the only previous study we are aware of, 78% of French physicians were opposed to the practice of UKD [18]. UKDs make an important contribution to the UK living donor programme via the UKLKSS and facilitate transplants for some of the most difficult to transplant patients on the waiting list. However, transplant professionals remain concerned about donor motivations, mental health issues and outcomes following UKD. It is crucial that robust data are provided to address this gap to either confirm or deny the apprehensions held by the transplant community. The longitudinal prospective study into UKDs’ outcomes will invariably help to fill this gap in due course.

Professional groups were not found to differ in their acceptance of UKD, which provides some baseline reassurance that units are working harmoniously in their approach towards UKDs. With regard to centre volume, this study has demonstrated that whilst higher volume centres report higher levels of acceptance for UKD, there are ongoing practical issues and more negative personal experiences. These somewhat mixed findings may be explained by the increased workload that UKD places on the existing living donor programme, leading to individuals feeling inadequately resourced, underprepared and overwhelmed. Fewer positive experiences with UKD candidates in higher volume centres may also reflect the larger number and broader range of individuals presenting as potential UKDs who then do not proceed for a variety of different reasons. Whilst the number of UKDs at each centre are known, the number of potential UKDs approaching each centre and the drop-out rates remains unclear. This is another area in which BOUnD will hopefully provide detailed data for the transplant community.

### Strengths and Limitations

The strengths of this study lie within its questionnaire tool which was devised and piloted with the specific research questions in mind. The study also sampled a large number and range of transplant staff and included every transplant centre in the UK. One limitation is that the questionnaire was not designed explore why participants held the views they expressed. A separate qualitative study has addressed some of these issues and is currently under peer review. Due to the questionnaire being distributed broadly across transplant centres and their professionals it was not possible to calculate the denominator in the population contacted. This introduces the potential for responder bias and a theoretical limitation regarding how representative this view is of the transplant professional population as a whole. There was also a potential for bias as individuals interested in, or with experience of, UKD may have been more likely to respond than those with little interest or experience.

### Conclusion

This study has demonstrated that whilst there is broad support of UKD as a practice, there are a number of potential barriers. These include a perception that UKDs are more time consuming and a need to exclude psychopathological motives prior to any medical tests being performed. There is ongoing uncertainty related to donor age and a feeling in higher volume centres that more training and resources are needed to facilitate UKD practices. The results from the prospective longitudinal study being conducted as part of BOUnD will provide a robust assessment of many of these factors and provide the transplant community with much needed data on this group of donors.

## Data Availability

The raw data supporting the conclusion of this article will be made available by the authors, without undue reservation.

## Abbreviations

ADCs, Altruistic Donor Chains; BOUnD, Barriers and Outcomes in Unspecified Donation; EAPM, European Association of Psychosomatic Medicine; HLA, Human Leucocyte Antigen; SKD, Specified Kidney Donation; SKDs, Specified Kidney Donors; UK, United Kingdom; UKD, Unspecified Kidney Donation; UKDs, Unspecified Kidney Donors; UKLKSS, UK Living Kidney Sharing Scheme.
