# Supplementary material for: Donating a Kidney to a Stranger: Are Healthcare Professionals Facilitating the Journey? Results From the BOUnD Study
Source: Transpl Int. 2023 May 30;36:11257. doi: 10.3389/ti.2023.11257 (PMC10261699; doi:10.3389/ti.2023.11257)
Supplement: Supplementary file 1 [file DataSheet1.PDF]

WITH PLYMOUTH UNIVERSITY

KING'S College LONDON

Guy's and St Thomas' NHS
NHS Foundation Trust

Understanding Barriers and Outcomes of Unspecified Kidney Donation

Transplant Professionals' Opinion Questionnaire

Why is this important?
We are conducting this study as part of a wider research programme funded by the National Institute for Health Research (NIHR) to evaluate the processes surrounding unspecified (altruistic) kidney donation.

We are interested in your personal views as a transplant professional regarding unspecified (non-directed altruistic) kidney donors and their process of donation. Your views are important to informing this research and may help improve clinical practice.

We estimate it will take you 10 to 15 minutes to complete.
The answers you give will be treated as confidential. To protect your anonymity demographic data will only be available to the study data manager and statistician. Data shared with the wider research team will be pooled to ensure individuals are not identifiable.

Please only complete this if you are a transplant professional or a senior trainee with a national training number.

Questionnaire Overview
The questionnaire aims to elicit the following:

- Some information about yourself and your own altruistic behaviour
- Donation practices in your transplant centre
- Your attitudes towards donation (deceased, living and altruistic)
- Your attitudes towards people who want to be "altruistic" donors
- Your views on the financial aspects of donation

Terminology
For the purposes of this questionnaire the following definitions will be used:

- **'Specified donors'** – individuals undergoing living donation where the recipient is known to the donor. These donors are also known as *directed* living donors.
- **'Unspecified donors'** – individuals who donate a kidney to someone they do not know, either as part of an altruistic living donor chain or directly to a stranger on the transplant waiting list. These are also known as altruistic or *non-directed altruistic* living donors.

Understanding Barriers and Outcomes of Unspecified Kidney Donation

Participant Information Sheet

Invitation and brief summary

You are invited to take part in a research study about unspecified (altruistic) kidney donation. We want to understand how healthcare professionals involved in living donor transplantation feel about the unspecified donation process and to hear about their experiences. There is some anecdotal evidence that some clinicians find unspecified donation difficult, either because they are uncomfortable with the concept or because of logistical pressures. However, no research has been carried out into the attitudes and experiences of transplant professionals towards unspecified donation in the UK.

This study is part of a larger project into unspecified donation, which is being funded by the NIHR Health Services and Delivery Research Programme. It will produce information that will inform the development of written resources for transplant professionals, and guidance to inform clinical practice and policy.

What would taking part involve?

- Taking part will involve completion of a questionnaire.
- The questionnaire will be sent by post or made available online.
- The questionnaire should take no more than 10-15 minutes to complete

Why have I been chosen?

You have been invited to take part because you have been identified as a transplant professional (nurse, nephrologist, transplant surgeon, transplant coordinator, mental health professional or other healthcare professional involved in kidney transplantation).

How have patients and the public been involved in this study?

Service users (previous unspecified donors) have helped develop the research topic and decide what research questions should be asked. One of them is a co-applicant who will continue to be involved in the study.

What are the possible benefits of taking part?

By taking part you will contribute to improved understanding of the process of unspecified donation in the UK. You will have contributed to new recommendations to improve services and to helpful resources for both donors and transplant professionals.

What are the possible disadvantages and risks of taking part?

If you feel uncomfortable with any of questions asked you do not have to complete the questionnaires.

What kind of questions will I be asked?

The questions will ask about your views, attitudes, practice and challenges of unspecified kidney donation, as well as methods of improving this.

Can I change my mind about taking part?
Absolutely, the study is entirely voluntary. However, as the questionnaire data is anonymous, once it has been submitted it is not possible to withdraw it.

Will my information be kept confidential?
Yes. All data will be anonymised. We will ensure that any published quotes contain no identifiable information.

What happens when the study is finished?
It is hoped that the study will be published and a copy of this final paper will be sent to you when it is complete. We will also send you copies of any new guidance developed as a result of this study.

What happens next?
If you would like to take part in this study then please complete the following questionnaire. Alternatively if you would prefer to complete this questionnaire online please call 0207 188 7188 (extension 52409) or email the study's manager (rebecca.gare@gstt.nhs.uk) who can provide you with a link.

Further information and contact details?
Thank you for expressing an interest in the study. If you have any questions or queries please contact Becki Gare (study manager) at rebecca.gare@gstt.nhs.uk. Alternatively you can contact the Chief Investigator, Professor Nizam Mamode on 0207 188 81543. If you have a concern about any aspect of this study, you should ask to speak to the researchers, who will do their best to answer your questions. If you have any general complaints you should contact the Patient Advice and Liaison Service (PALS) at Guy's and St Thomas' NHS Foundation trust (Tel: 02071888801; Email: pals@gstt.nhs.uk). If you have any concerns about the way in which the study has been conducted, please contact the Trust's Research and Development Department (R&D) via the hospital's switchboard 02071887188.

I confirm that I have read and understood the above Participant Information Sheet and I am happy to proceed with the questionnaire:

1. Date: ___________________ 2. Yes □ No □

Thank you very much for reading this information sheet and for considering our research study.

This project was funded by the National Institute for Health Research HS&DR Project (project number 13/54/54)

NHS
National Institute for Health Research

<u>BOUnD Questionnaire</u>

Transplant Professionals' Opinion

Participant Demographics

3. Please specify your role within your transplant team:

Administration team □
Inpatient Nurse □
Outpatient Nurse □
Nurse co-ordinator (donor / recipient) □
Physician (consultant) □
Physician (senior trainee with a national training number) □
Surgeon (consultant) □
Surgeon (senior trainee with a national training number) □
Other Healthcare Professional □

3a) Which Transplant centre/s do you work in?

3b) What is your gender?
Female □ Male □

3c) What is your age range?
18-24 □ 25-34 □ 35-44 □ 44-55 □ 55+ □

3d) Do you consider yourself as part of a minority ethnic group?
Yes □ No □ Prefer not to answer □

3e) Would you describe yourself as a spiritual person?
Yes □ No □ Prefer not to answer □

3f) Would you describe yourself as a religious person?
Yes □ No □ Prefer not to answer □

3g) Are you involved directly with unspecified (non-directed altruistic) kidney donors?
Yes □ No □ Prefer not to answer □

If YES:
i) How many years have you worked with unspecified (non-directed altruistic) kidney donors?
<2 years □ 2-5 years□ 5-10 □ >10 □

ii) How many unspecified (non-directed altruistic) kidney donors have you had contact with during your career?
<5 □ 5-10□ 11-20 □ >20 □ I don't know □

3h) Do you give or have you ever given blood?
Yes □ No □ Not eligible □ Prefer not to answer □

3i) Are you on the bone marrow register?
Yes □ No □ Not eligible □ Prefer not to answer □

3j) Are you on the organ donor register?
Yes □ No □ Not eligible □ Prefer not to answer □

3k) Do you give money, goods or your time to charity on a regular basis?
Yes □ No □ Prefer not to answer □

3l) Do you do any voluntary work?
Yes □ No □ Prefer not to answer □

Clinical practice in unspecified (non-directed altruistic) kidney donation

Please answer the following questions about the Centre in which you work…

4. My centre has an **upper age limit** for unspecified (non-directed altruistic) kidney donation

Yes □ No □ Not sure □

If YES, the upper age limit is:__________ years

5. My centre has a **lower age limit** for unspecified (non-directed altruistic) kidney donation

Yes □ No □ Not sure □

If YES, the lower age limit is:__________ years

6. My centre has a specific living donor co-ordinator in charge of the unspecified (non-directed altruistic) kidney donation programme?

Yes □ No □ Not sure □

7. All decisions about potential unspecified (non-directed altruistic) donors are made in an MDT setting?

Yes □ No □ Not sure □

8. Individuals making initial enquiries regarding unspecified (non-directed altruistic) donation receive an enthusiastic response when contacting my centre

Strongly agree □
Agree □
Neither agree nor disagree □
Disagree □
Strongly disagree □

9. Sufficient clinical facilities are currently available in my centre to allow unspecified (non-directed altruistic) donation

Strongly agree □
Agree □
Neither agree nor disagree □
Disagree □
Strongly disagree □

10. There is currently sufficient support at a national level to help my centre with any queries or problems regarding unspecified (non-directed altruistic) donation

Strongly agree □
Agree □
Neither agree nor disagree □
Disagree □
Strongly disagree □

11. Sufficient staff training is currently available at my centre to allow unspecified (non-directed altruistic) donation.

Strongly agree □
Agree □
Neither agree nor disagree □
Disagree □
Strongly disagree □

12. A national protocol for unspecified (non-directed altruistic) kidney donation would be beneficial for selecting potential donors

Strongly agree □
Agree □
Neither agree nor disagree □
Disagree □
Strongly disagree □

13. Unspecified (non-directed altruistic) kidney donation should occur only in centres with a 'specialist interest' in this aspect of transplantation

Strongly agree □
Agree □
Neither agree nor disagree □
Disagree □
Strongly disagree □

14. In my centre all unspecified (non-directed altruistic) donors have a mental health assessment prior to donation

Yes □ No □ Not sure □

If YES, at what stage does this typically occur?

A. Before any medical tests are performed □
B. Before the donor sees a nephrologist □
C. Before the donor sees a surgeon □
D. Before the independent assessment □
E. Immediately before donation □
F. Don't know □

15. In **<u>your opinion</u>**, do you think every unspecified (non-directed, altruistic) donor should have a mental health assessment prior to donation?

Yes □ No □ Not sure □

If YES, at what stage should this occur?

A. Before any medical tests are performed □
B. Before the donor sees a nephrologist □
C. Before the donor sees a surgeon □
D. Before the independent assessment □
E. Immediately before donation □
F. Don't know □

Of those individuals making initial contact with a transplant centre to request further information, what percentage would you **estimate** proceed to donate as an unspecified (non-directed altruistic) donor?

16. In your centre

<10% □ 10-20% □ 20-30% □ 30-40% □ 40-50% □
50-60% □ 60-70% □ 80-90% □ >90% □

17. In the UK

<10% □ 10-20% □ 20-30% □ 30-40% □ 40-50% □
50-60% □ 60-70% □ 80-90% □ >90% □

Your views regarding different types of kidney donation

We would like to know more about your general attitudes towards different types of organ donation. Please state how much you agree or disagree with the following statements. Presume that there are no contraindications to being a donor.

18. I am comfortable with the idea of donating my organs **after death**

Strongly agree	□
Agree	□
Neither agree nor disagree	□
Disagree	□
Strongly disagree	□

19. Which of the following best describes how you personally feel about organ donation **after death**?

□ I would definitely want to donate **some or all** of my organs after death if possible

□ I would consider donating **some or all** of my organs after death

□ I don't know if I want to donate **some or all** of my organs after death

□ I definitely don't want to donate **some or all** of my organs after death

20. I am comfortable with the idea of being a living kidney donor for **someone I know**.

Strongly agree	□
Agree	□
Neither agree nor disagree	□
Disagree	□
Strongly disagree	□

21. Which of the following best describes how strongly you feel about being a living (specified) donor for **someone you know**?

□ I would definitely want to become a living donor if a close friend / family member were in need
□ I would consider becoming a living donor if a close friend / family member were in need
□ I don't know if I would consider become a living donor if a close friend /family member were in need
□ I definitely would not want to become a living donor if a close friend / family member were in need

22. I am comfortable with the idea of being a living kidney donor for **someone I do not know.**

Strongly agree □
Agree □
Neither agree nor disagree □
Disagree □
Strongly disagree □

23. Which of the following best describes how you personally feel about being an **unspecified (non-directed altruistic) kidney donor** at some point in your life:

- □ I would definitely want to become an altruistic kidney donor
- □ I would consider becoming an altruistic kidney donor
- □ I don't know if I would consider becoming an altruistic kidney donor
- □ I definitely would not want to become an altruistic kidney donor

Working with unspecified (non-directed altruistic) kidney donors

24. I am confident dealing with people wishing to become unspecified (non-directed altruistic) kidney donors

Strongly agree □
Agree □
Neither agree nor disagree □
Disagree □
Strongly disagree □

25. My experience with people wishing to become unspecified (non-directed altruistic) donors has been generally positive

Strongly agree □
Agree □
Neither agree nor disagree □
Disagree □
Strongly disagree □

26. I am comfortable with unspecified (non-directed altruistic) donors undergoing major surgery

Strongly agree □
Agree □
Neither agree nor disagree □
Disagree □
Strongly disagree □

27. I believe it is possible for individuals to be motivated purely by the desire to help others

Strongly agree	□
Agree	□
Neither agree nor disagree	□
Disagree	□
Strongly disagree	□

28. I believe unspecified (non-directed altruistic) living kidney donors make balanced decisions when choosing/deciding whether to donate or not

Strongly agree	□
Agree	□
Neither agree nor disagree	□
Disagree	□
Strongly disagree	□

29. I am worried about the potential long-term effects of unspecified (non-directed altruistic) donation on the physical health of the donor

Strongly agree	□
Agree	□
Neither agree nor disagree	□
Disagree	□
Strongly disagree	□

30. I am worried about the potential long-term effects of unspecified (non-directed altruistic) donation on the psychological health of the donor

Strongly agree	□
Agree	□
Neither agree nor disagree	□
Disagree	□
Strongly disagree	□

31. I am worried that unspecified (non-directed altruistic) living donation is potentially a burden for the donor's family

Strongly agree	□
Agree	□
Neither agree nor disagree	□
Disagree	□
Strongly disagree	□

32. I am worried the unspecified (non-directed altruistic) donor may regret their decision to donate in the future

- Strongly agree □
- Agree □
- Neither agree nor disagree □
- Disagree □
- Strongly disagree □

What do you think potential unspecified (non-directed altruistic) donors are motivated by?

33. Personal psychological benefit
- Strongly agree □
- Agree □
- Neither agree nor disagree □
- Disagree □
- Strongly disagree □

34. Change in social status
- Strongly agree □
- Agree □
- Neither agree nor disagree □
- Disagree □
- Strongly disagree □

35. Religious or spiritual beliefs
- Strongly agree □
- Agree □
- Neither agree nor disagree □
- Disagree □
- Strongly disagree □

36. Civic duty and social responsibility
- Strongly agree □
- Agree □
- Neither agree nor disagree □
- Disagree □
- Strongly disagree □

37. Personal psychological ill-health
- Strongly agree □
- Agree □
- Neither agree nor disagree □
- Disagree □
- Strongly disagree □

How often do the following factors prevent potential unspecified (non-directed altruistic) donors from donating?

38. Medical fitness
Very frequently □ Frequently □ Sometimes □ Rarely □ Never □Not sure □

39. Mental health fitness
Very frequently □ Frequently □ Sometimes □ Rarely □ Never □Not sure □

40. Anxiety about donation / Change of mind
Very frequently □ Frequently □ Sometimes □ Rarely □ Never □Not sure □

41. Lack of family support
Very frequently □ Frequently □ Sometimes □ Rarely □ Never □Not sure □

42. Negative attitudes of transplant professionals
Very frequently □ Frequently □ Sometimes □ Rarely □ Never □Not sure □

43. Negative attitudes of community professionals (e.g. GP, practice nurse, etc)
Very frequently □ Frequently □ Sometimes □ Rarely □ Never □Not sure □

44. Prolonged duration of the donation work-up
Very frequently □ Frequently □ Sometimes □ Rarely □ Never □Not sure □

45. Financial concerns for the potential donor
Very frequently □ Frequently □ Sometimes □ Rarely □ Never □Not sure □

Please state your views, comparing unspecified (non-directed altruistic) kidney donors with other living kidney donors.

46. How are unspecified (non-directed altruistic) donors treated during the donation process compared to other living donors?

Much better □
Better □
The same □
Worse □
A lot worse □
Not sure □

47. Unspecified (non-directed altruistic) donors receive less support after donation

Strongly agree □
Agree □
Neither agree nor disagree □
Disagree □
Strongly disagree □

48. I feel there should be an upper age limited for unspecified (non-directed altruistic) donation

Yes □ No □ Not sure □

48 a) If YES, this age should be: _______ years

49. I feel there should be a lower age limit for unspecified (non-directed altruistic) donation

Yes □ No □ Not sure □

49 a) If YES, this age should be: _______ years

50. I think many people wishing to be unspecified (non-directed altruistic) kidney donors are likely to have a history of mental health problems

Strongly agree □
Agree □
Neither agree nor disagree □
Disagree □
Strongly disagree □

51. I think many people wishing to be unspecified (non-directed altruistic) kidney donors are more likely to be risk-takers who do not fully consider the consequences of their actions

Strongly agree □
Agree □
Neither agree nor disagree □
Disagree □
Strongly disagree □

Resources relevant to unspecified (non-directed altruistic) donation

Compared to other living kidney donors, **BEFORE** donation, potential unspecified (non-directed altruistic) donors:

52. Have a higher dropout rate

Strongly agree □
Agree □
Neither agree nor disagree □
Disagree □
Strongly disagree □

53. Are more time consuming for transplant professionals

Strongly agree □
Agree □
Neither agree nor disagree □
Disagree □
Strongly disagree □

54. Need a greater number of assessments or investigations compared with specified living donors

Strongly agree □
Agree □
Neither agree nor disagree □
Disagree □
Strongly disagree □

Compared with other living kidney donors, **AFTER** donation, unspecified (non-directed altruistic) donors are:

55. More likely to seek medical help from transplant units regarding donation related issues

Strongly agree □
Agree □
Neither agree nor disagree □
Disagree □
Strongly disagree □

56. More likely to seek mental health help regarding donation related issues

Strongly agree □
Agree □
Neither agree nor disagree □
Disagree □
Strongly disagree □

57. More likely to seek medical help from transplant units regarding non-donation related issues compared to specified donors

- Strongly agree □
- Agree □
- Neither agree nor disagree □
- Disagree □
- Strongly disagree □

58. Thank you for completing our questionnaire. If you have anything additional you wish to say then please do so below…

SDC 2, Materials and Methods: Details of questionnaire development

The questionnaire design process was undertaken by a multi-professional panel of transplant clinicians (including nephrologists, transplant surgeons and clinical psychologists), academic psychologists, a statistician and previous UKDs. This was in order to obtain a broad, balanced and multidimensional perspective on issues related to UKD.

The topics to be covered by the questionnaire were informed by focus groups undertaken with service users (including those who became UKDs, those who had self-withdrawn from the process and those withdrawn by the clinical team) and transplant professionals. The focus groups were coordinated by clinical and academic psychologists. Thirty-four transplant professionals participated in four focus groups, representing eight separate professional roles including, transplant surgeons, nephrologists, ward nurses, independent assessors and transplant coordinators. A thematic analysis was performed on the focus group data, from which the key topics were extracted.

Questionnaire items were written to account for an average reading age and to minimise ambiguity in their interpretation. Each question was provided with a list of options to select from or with a Likert scale on which to answer. The first draft of the questionnaire was reviewed and modified by the research team who had experience in questionnaire writing and validation. The questions then underwent validity and reliability testing prior to finalisation in order to minimise the possibility of incorrect conclusions being made from the data. Ten transplant professionals were involved in developing and finalising the questionnaire. Three participated in a cognitive 'think aloud' face validation exercise to identify any questionnaire items that were ambiguous or misleading. Subsequently, seven professionals completed the questionnaire to provide baseline answers and estimates of completion time. Five completed it again two weeks later to assess test-retest reliability.

SDC 3, Materials and Methods: Psychometrics for the "Acceptance of UKD" score

The following items were collated to form the "Acceptance of UKD" scale. These were answered on a 5-point Likert scale (strongly agree to strongly disagree).

	Question	Label	N	Mean	SD	Min	Max
1	Individuals making initial enquiries regarding unspecified (non-directed altruistic) donation receive an enthusiastic response when contacting my centre	cenenthu	151	0.9	0.8	0	3
2	Sufficient clinical facilities are currently available in my centre to allow unspecified (non-directed altruistic) donation	cenfacil	151	0.9	1.0	0	4
3	There is currently sufficient support at a national level to help my centre with any queries or problems regarding unspecified (non-directed altruistic) donation	cennatsupp	151	1.2	0.8	0	4
4	Sufficient staff training is currently available at my centre to allow unspecified (non-directed altruistic) donation.	centrain	152	1.3	1.0	0	4
5	I am confident dealing with people wishing to become unspecified (non-directed altruistic) kidney donors	ukdconf	151	0.7	0.8	0	4
6	My experience with people wishing to become unspecified (non-directed altruistic) donors has been generally positive	ukdposv	152	0.9	0.7	0	3
7	I am comfortable with unspecified (non-directed altruistic) donors undergoing major surgery	ukdsurg	152	0.9	0.7	0	3

Inter-item correlations

	cenenthu	cenfacil	cennatsupp	centrain	ukdconf	ukdposv	ukdsurg
**cenenthu**	1						
**cenfacil**	0.34	1					
**cennatsupp**	0.39	0.46	1				
**centrain**	0.25	0.62	0.42	1			
**ukdconf**	0.39	0.37	0.48	0.37	1		
**ukdposv**	0.48	0.26	0.38	0.32	0.65	1	
**ukdsurg**	0.41	0.4	0.42	0.32	0.64	0.67	1

The first principle component of the correlation matrix explained 52% of the variance in responses to the acceptance items. Assuming the first principle component represents acceptance, this indicates that factors other than acceptance explain relatively little additional variation in responses. Parallel analysis indicated that the items are likely to form a unidimensional scale. The plot below shows that only the eigenvalues of the first principle components (solid line) are above those expected by chance (dashed line).

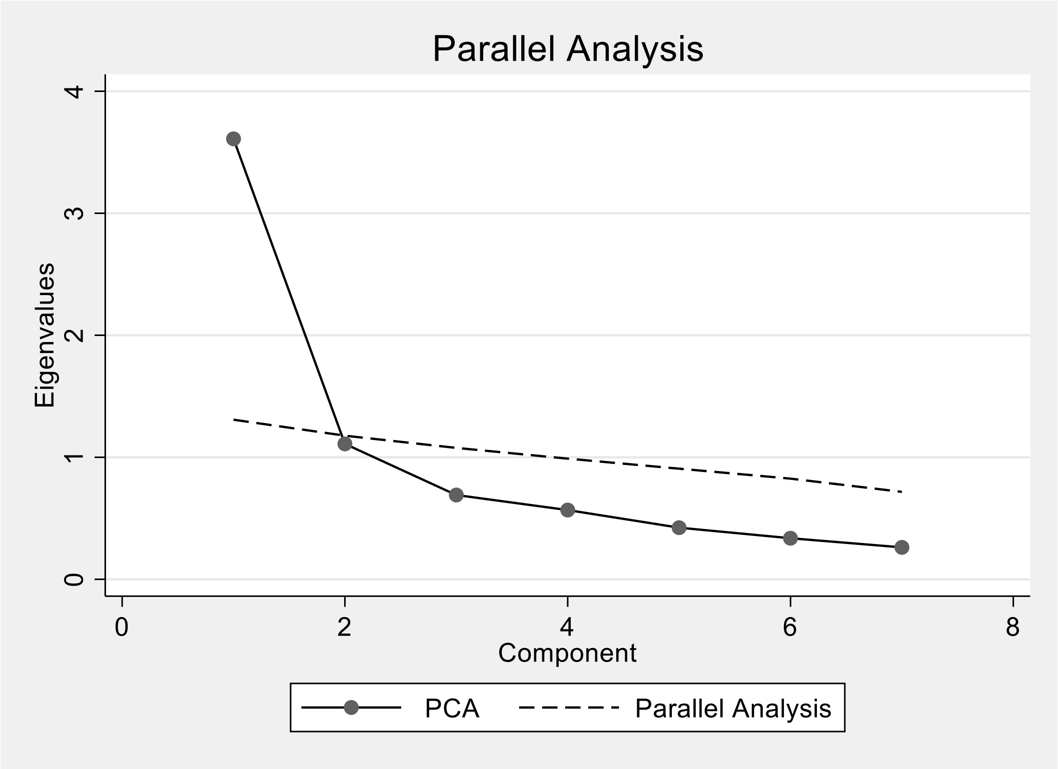

Reliability assessed using Cronbach's alpha indicated that forming the items into a scale produced a scale within the bounds of what is generally considered acceptable (i.e. alpha >0.7).

Item	N	Sign	Item-total correlation	alpha
cenenthu	151	+	0.60	0.76
cenfacil	151	+	0.62	0.76
cennatsupp	151	+	0.67	0.75
centrain	152	+	0.59	0.76
ukdconf	151	+	0.70	0.74
ukdposv	152	+	0.69	0.74
ukdsurg	152	+	0.72	0.73
Total-score				0.78

The total acceptance score was calculated where higher scores indicate greater acceptance of UKD. To account for differing response categories across items the scale of the score was standardised with the mean for the sample set at 50 and the standard deviation of 10. This is an arbitrary scale but allows for comparisons across groups within the sample. The distribution was approximately normal with mild positive skew.

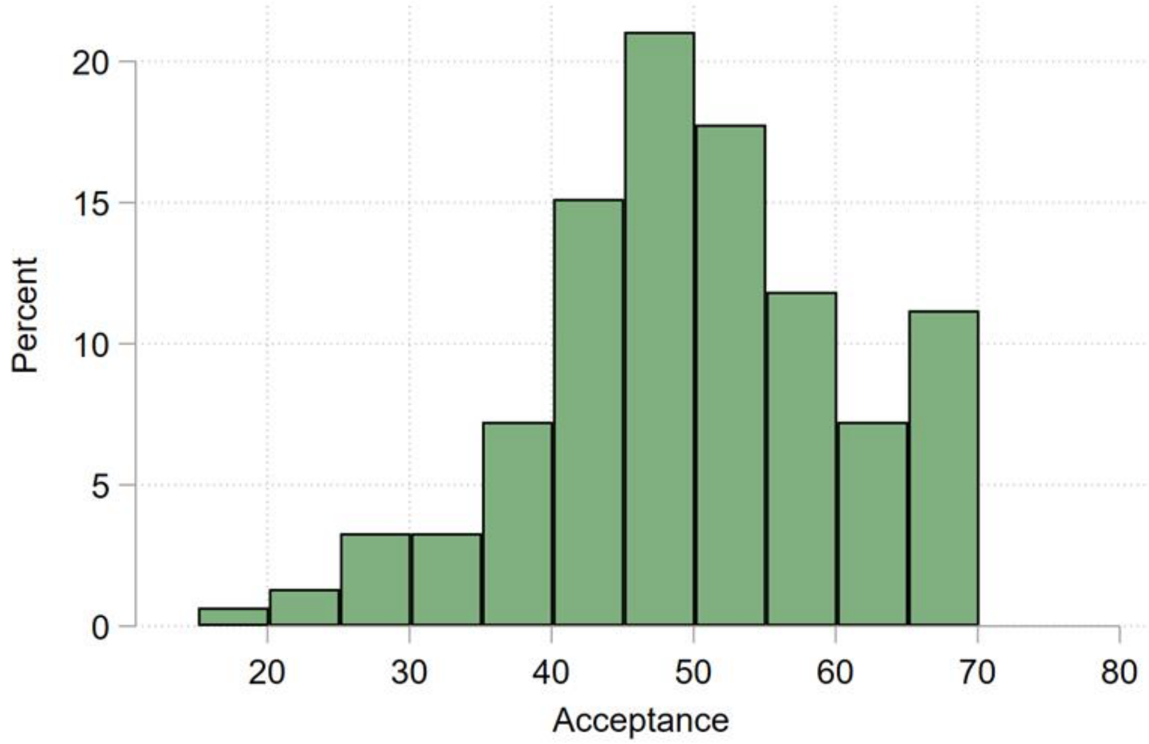

Crude means across role groups

	N	Mean	SD
Co-ordinator or nurse	45	49.1	10.2
Physician/Surgeon	69	50.3	11.7
Other	38	50.5	8.4
Total	152	50.0	10.4

Adjusted means across groups were not significant and are provided in the main manuscript (figure 4). Correlations between support and other variables were calculated and are also provided in the main manuscript (table 4).
